# HDAC inhibition activates the apoptosome via *Apaf1* upregulation in hepatocellular carcinoma

**DOI:** 10.1186/s40001-016-0217-x

**Published:** 2016-06-24

**Authors:** Reena Buurman, Maria Sandbothe, Brigitte Schlegelberger, Britta Skawran

**Affiliations:** Institute of Human Genetics, Hannover Medical School, Carl-Neuberg-Straße 1, 30625 Hannover, Germany

**Keywords:** Hepatocellular carcinoma, Acetylation, Histone deacetylases, Apoptosis, Trichostatin A

## Abstract

**Background:**

Histone deacetylation, a common hallmark in malignant tumors, strongly alters the transcription of genes involved in the control of proliferation, cell survival, differentiation and genetic stability. We have previously shown that *HDAC1*, *HDAC2*, and *HDAC3* (*HDAC1*–*3*) genes encoding histone deacetylases 1–3 are upregulated in primary human hepatocellular carcinoma (HCC). The aim of this study was to characterize the functional effects of HDAC1–3 downregulation and to identify functionally important target genes of histone deacetylation in HCC.

**Methods:**

Therefore, HCC cell lines were treated with the histone deacetylase inhibitor (HDACi) trichostatin A and by siRNA-knockdown of *HDAC1*–*3*. Differentially expressed mRNAs were identified after siRNA-knockdown of *HDAC1*–*3* using mRNA expression profiling. Findings were validated after siRNA-mediated silencing of HDAC1–3 using qRTPCR and Western blotting assays.

**Results:**

mRNA profiling identified apoptotic protease-activating factor 1 (*Apaf1*) to be significantly upregulated after HDAC inhibition (HLE siRNA#1/siRNA#2 *p* < 0.05, HLF siRNA#1/siRNA#2 *p* < 0.05). As a component of the apoptosome, a caspase-activating complex, Apaf1 plays a central role in the mitochondrial caspase activation pathway of apoptosis. Using annexin V, a significant increase in apoptosis could also be shown in HLE (siRNA #1 *p* = 0.0034) and HLF after siRNA against *HDAC1*–*3* (Fig. 3a, b). In parallel, caspase-9 activity was increased after siRNA-knockdown of *HDAC1*–*3* leading to enhanced apoptosis after HDAC inhibition (Fig. 3c, d).

**Conclusions:**

The present data show that siRNA-knockdown of *HDAC1*–*3* plays a major role in mediating apoptotic response to HDAC inhibitors through regulation of Apaf1.

## Background

Hepatocellular carcinoma (HCC) represents the fourth most common malignant tumor with more than 1 million persons affected per year worldwide [1, 2]. HCC is associated with a very poor prognosis (http://www-dep.iarc.fr/) [3, 4] and only limited treatment options are available. Therefore, new effective therapeutic strategies are urgently needed.

Currently, cancer development is regarded as an interaction of genetic, genomic and epigenetic alterations [5–9]. The histone modification, i.e. acetylation, of lysine residues is an important secondary modification responsible for chromatin remodeling and is controlled by histone acetyltransferases (HATs) and histone deacetylases (HDACs). The deacetylation of chromatin results in the formation of a higher order, i.e. condensation of chromatin, leading to the repression of transcription of many genes involved in multiple cellular functions. Notably, epigenetic modifications are reversible and can be targeted by new drugs like DNA methyltransferase and HDAC inhibitor (HDACi), drugs that have shown efficacy in clinical phase I/II studies (http://www.clinicaltrials.gov) [10]. HDACi are found to have an anti-cancer function in many different tumors [11, 12].

We have previously shown that *HDAC1*–*3* are upregulated in primary human HCC [13]. Therefore, we hypothesized that the altered expression of genes due to chromatin remodeling may play a fundamental role in hepatocarcinogenesis. We hence induced histone acetylation by HDACi or siRNA silencing of *HDAC1*–*3* to identify functionally important target genes. Upon increasing histone acetylation, the apoptotic protease-activating factor 1 (Apaf1), a major regulator of apoptosis, was reactivated.

## Methods

### Primary tissue

Analysis was carried out based on the re-evaluation of pseudonymized tumor specimens of 23 patients with HCC treated at Hannover Medical School (MHH) and taken from the archive of the Institute of Pathology at the MHH (Germany) [13]. The local Ethics Committee (“Ethikkommission der Medizinischen Hochschule Hannover”, head: Prof. Dr. H.D. Tröger) approved the application to retrospectively use the samples in this study (left over from diagnostic procedures) that had been irreversibly unlinked from the source, rendering them anonymous, and thus exempting them from IRB review, waiving the consent requirement due to no legal or ethical concerns (Ethics Statement: No. 2208–2014).

### Cell culture, HDAC inhibition and transfection

HCC cell lines HLE [14] and HLF [14] (kindly provided by Professor Nam-Ho Huh, Department of Cell Biology, Graduate School of Medicine, Dentistry and Pharmaceutical Sciences, Okayama University, Okayama, Japan) were treated with trichostatin A (TSA) or transfected with siRNA against *HDAC1*–*3* as previously described [13]. siRNA#1 and #2 are different mixtures of siRNAs against HDAC1, HDAC2 and HDAC3 provided by Qiagen, Hilden, Germany (siRNA#1 = Hs_HDAC1_1, Hs_HDAC2_3, Hs_HDAC3_10; siRNA#2 = Hs_HDAC1_6, Hs_HDAC2_1, Hs_HDAC3_9).

### Expression analyses

Microarray analyses were done as previously described with Whole Human Genome Oligo Microarray Kit 4 × 44 k (Agilent) [13]. The array analysis was performed by a paired *t* test of treated against untreated cells with a corrected (Benjamini–Hochberg) *p* value of 0.1. The Agilent GeneSpring GX Data Analysis Software was used for bioinformatic analysis. mRNAs of interest were validated by qRTPCR as previously described [13] with Taqman Assay [Hs00559421_m1, amplicon spans exon 10 and 11 with a length of 112 bp (Applied Biosystems)] and Western blot using antibody against Apaf1 [#8723 Cell Signaling/NEB Danvers, MA, USA, Antibody ID: AB_10829610 from http://www.antibodyregistry.org, used 1:1000, blocking with 3 % Slim Fast chocolate (Allpharm)].

### Assays to determine apoptosis and caspase-9 activity

To detect apoptotic cells, both adherent and floating cells were collected and washed twice with PBS. Cells were resuspended in 1× binding buffer (Becton–Dickinson) and stained with 5 µL annexinV-APC (Becton–Dickinson) and 5 µL 7-AAD (Becton–Dickinson) for 15 min in the dark. Samples were analyzed with the FACSCalibur flow cytometer (Becton–Dickinson). Data analysis was performed using CellQuest Pro software (Becton–Dickinson).

To detect viable cells, the activity of the mitochondrial dehydrogenase was determined using the Cell Proliferation Reagent WST-1 in 96-well format (Roche, Mannheim, Germany). To determine caspase-9 activity, Caspase-Glo^®^ 9 Assay (G8210, Promega) was used. Absorption was measured using the Synergy 2 Multi-Mode Microplate Reader (BioTek).

### Statistics

For statistical analysis, GraphPad Prism version 5.02 for Windows was used. Each assay (except for microarrays) was performed three times in biologically independent assays. 1-way ANOVA with Dunnett’s multiple comparison test was performed. Statistics are given as mean ± standard deviation. Asterisks are related to the following *p* values in all experiments: *p = 0.01–0.05 significant, **p = 0.001–0.01 very significant, and ***p < 0.001 extremely significant.

## Results

As recently published by Buurman et al. [13], *HDAC1*–*3* are upregulated in human primary HCC. To characterize the functional effects of deregulated *HDAC1*–*3*, HCC cell lines HLE, HLF, HepG2 and Huh-7 were treated with HDACi TSA. To rule out such unspecific and toxic side effects of HDACs by inhibitors like TSA in the two cell lines HLE and HLF, siRNA-knockdown of combined *HDAC1*–*3* was performed.

Aiming to determine the influence of increased histone acetylation on mRNA expression in HCC, we investigated global mRNA expression by microarray analyses in HCC cell lines treated with specific siRNA against *HDAC1*–*3* for 48 h.

This led to significant differences in gene expression between treated and untreated cells. *Apaf1* showed the most pronounced differential expression levels compared to untreated cells. Since Apaf1 is a central protein of the intrinsic apoptotic pathway and the core molecule in the formation of the apoptosome, a caspase-activating complex, this gene was investigated further.

**Fig. 1:**
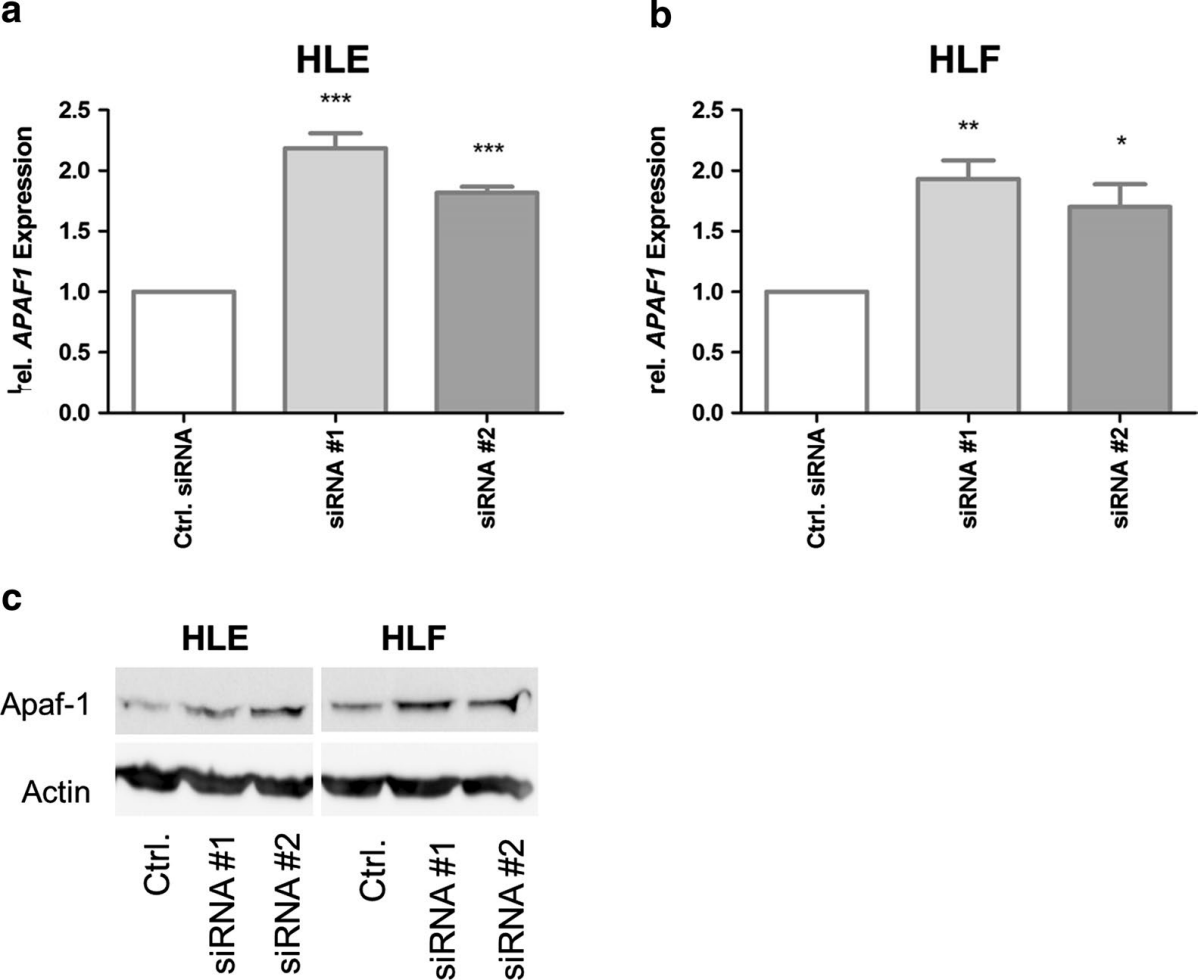
Expression of *Apaf1* after siRNA transfection against *HDAC1*–*3* in HLE and HLF. 48 h after transfection of siRNA against *HDAC1*–*3*, the HCC cell lines **a** HLE and **b** HLF showed a significant increased expression of Apaf1 measured by qRTPCR. **c** Western blot analysis of Apaf1 after siRNA showed a significant increase in *Apaf1* expression compared with controls. Values are mean ± SEM, *n* = 3. ***p < 0.001, **p < 0.01, *p ≤ 0.05, ANOVA plus *Dunnett*’s posttest

As shown in Fig. 1a, b, qRTPCR confirmed the result of the array analyses and showed a systematic increase in *Apaf1* expression after siRNA treatment against *HDAC1*–*3* (HLE siRNA#1/siRNA#2 *p* < 0,05, HLF siRNA#1/siRNA#2 *p* < 0,05). HLE and HLF showed increased Apaf1 expression using siRNA#1 about 118/93 % in comparison to the controls and using siRNA#2 about 81/70 %. Furthermore, expression of Apaf1 was analyzed by Western blot analyses (Fig. 1c) in HLE and HLF cell lines after siRNA treatment against *HDAC1*–*3*. An increase in Apaf1 protein expression was seen after histone deacetylation inhibition, substantiating also the result of the microarray analyses.

**Fig. 2:**
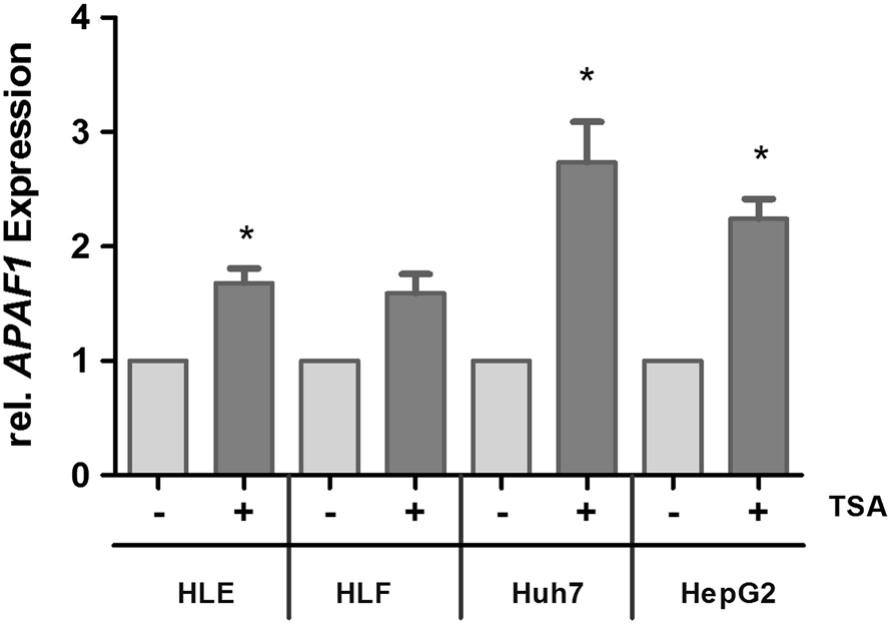
Expression of *Apaf1* after 12-h treatment with the HDACi TSA. Expression of *Apaf1* after 12-h treatment with the HDACi TSA in the HCC cell lines HLE, HLF, Huh7 and HepG2 measured by qRTPCR. After treatment, *Apaf1* showed an increased expression compared with controls. Values are mean ± SEM, *n* = 3, *p ≤ 0.05, ANOVA plus *Dunnett*’s posttest

Moreover, qRTPCR was performed focusing on *Apaf1* in the four HCC cell lines HLE, HLF, HepG2 and Huh-7 which were incubated with HDACi TSA for 12 h to validate the results from previous experiments. As shown in Fig. 2, qRTPCR confirmed the result of siRNA treatment against *HDAC1*–*3* and showed an increase in *Apaf1* expression after HDACi treatment. HLE, Huh7 and HepG2 showed an increased *Apaf1* expression with a significance of *p* = 0.01–0.05 (HLE 68 %, HLF 59 %, Huh7 173 %, HepG2 = 124 % increase).

**Fig. 3:**
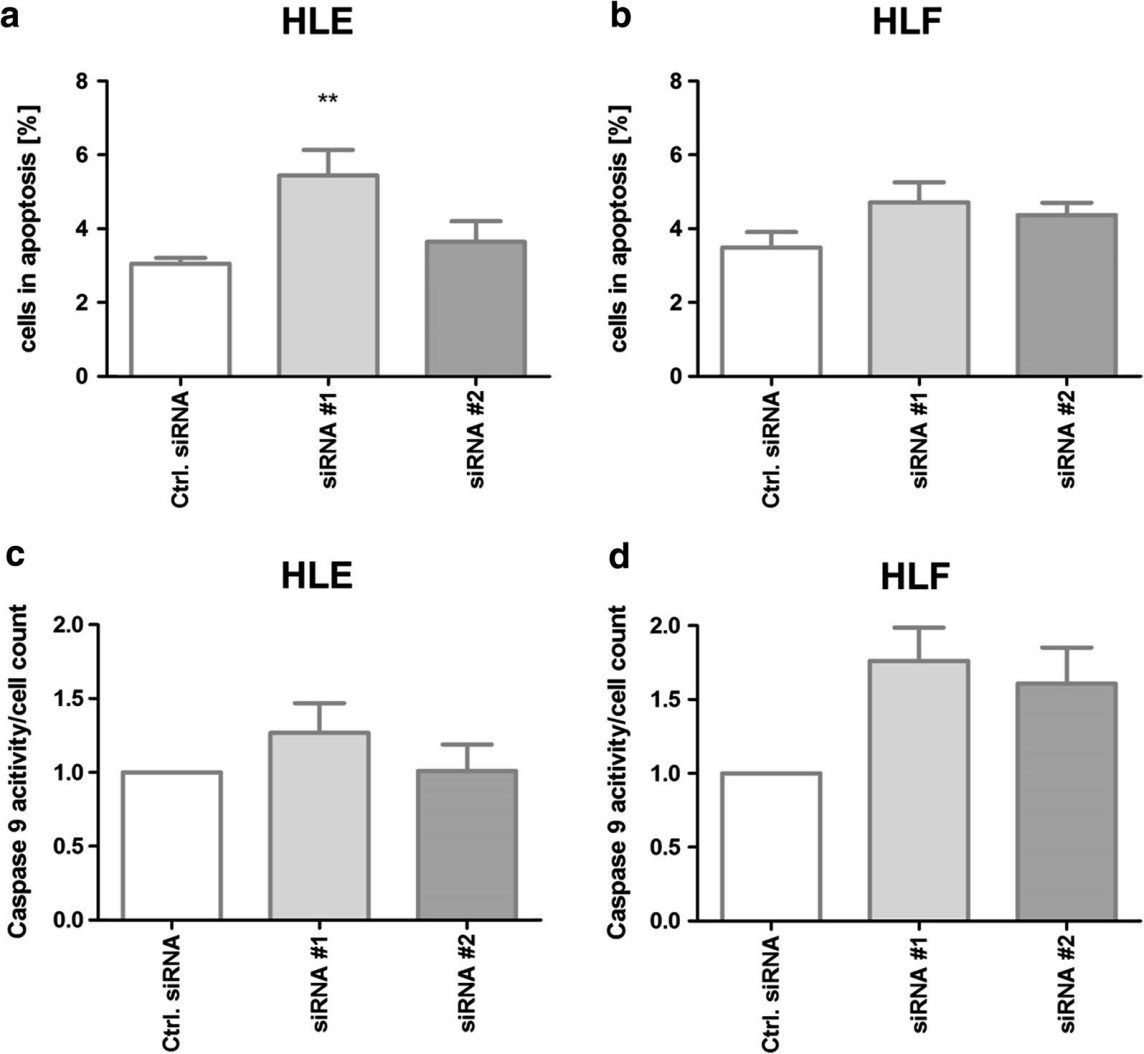
Apoptosis after siRNA transfection against *HDAC1*–*3.* Apoptosis measured by flow cytometry using annexin V staining in HLE **a** and HLF **b** is increased in both cell lines. Caspase-9 activity in the HCC cell lines **c** HLE and **d** HLF normalized to cell count after 48 h showed a significant increase in caspase-9 activity in both cell lines compared with controls. Values are mean ± SEM, *n* = 3. **p < 0.01, *p = <0.05, ANOVA plus *Dunnett*’s posttest

To investigate the effect of histone acetylation and upregulation of Apaf1 on apoptosis induction, apoptosis and proliferation were measured 48 h after siRNA transfection against *HDAC1*–*3*. In a previous study [13], it could be shown that acetylation was increased by more than 50 % and a significant increase in caspase 3/7 activity of more than 50 % than in controls. Using annexin V, a significant increase in apoptosis could also be shown in HLE [siRNA#1 80 %, siRNA#2 20 % increase (siRNA #1 *p* = 0.0034)] and HLF (siRNA#1 38 %, siRNA#2 28 % increase) after siRNA against *HDAC1*–*3* (Fig. 3a, b). In parallel, both HCC cell lines showed an increase of caspase 9 activity after 48 h compared with controls (Fig. 3c, d). The increase was between 26 and 76 % with exception of siRNA#2 in HLE.

**Fig. 4:**
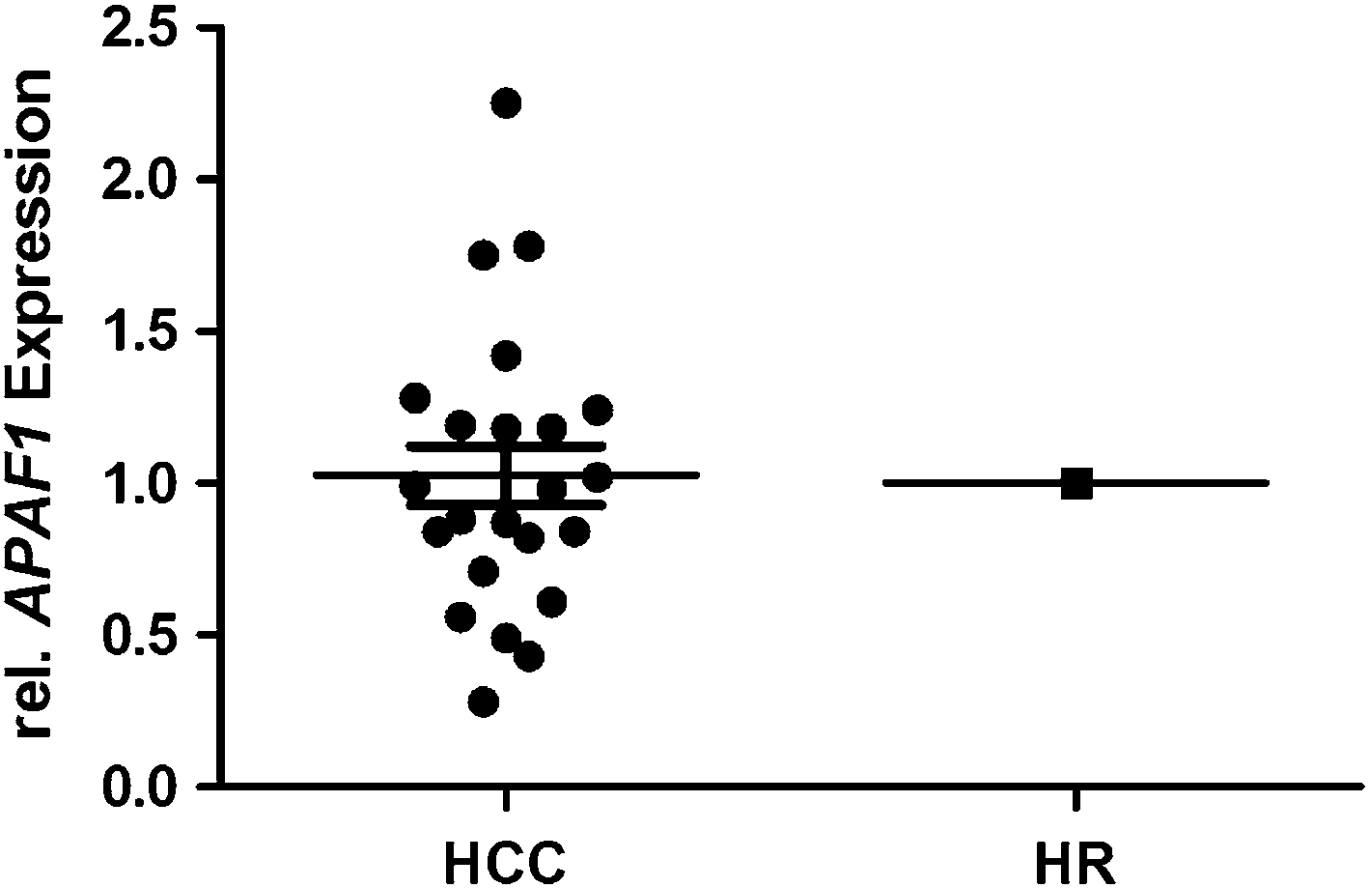
Expression of *APAF1* measured by qRTPCR in 23 primary HCC compared to a human reference. Primary human HCC showed reduced expression of *Apaf1* compared to a human reference

To confirm the clinical relevance of the deregulation of *Apaf1,* 23 primary HCC were investigated using qRTPCR. Compared to a human reference, *Apaf1* expression was reduced in more than half of the primary HCC (Fig. 4).

## Discussion

Histone deacetylation represents an important epigenetic modification in cancer development and is responsible for condensation of chromatin and transcriptional silencing in the respective genomic regions. In a previous study, we have shown that *HDAC1*–*3* are consistently upregulated in HCC [13].

Transcriptional repressors such as class I HDACs are receiving growing attention as potential therapeutic targets in human malignancies [15]. Several HDACi have been identified that drive tumor cells into growth arrest, differentiation, and apoptotic cell death [16]. Meanwhile several HDAC-specific inhibitors are being tested in preclinical and clinical trials [15].

In a previous study, we have shown that treatment of HCC cell lines with the HDACi TSA or with specific siRNA against *HDAC1*, *2* and *3* led to increased histone acetylation [13]. Using gene expression profiling after specific downregulation of *HDAC1*, *2* and *3* in HCC cell lines HLE and HLF, we observed an upregulation of *Apaf1*. *Apaf1* showed the most pronounced differential expression levels compared to untreated cells. This gene plays a central role in the mitochondrial caspase activation pathway of apoptosis. Indeed, after siRNA treatment against *HDAC1*–*3*, Apaf1 expression was increased, caspase-9 was activated and apoptosis induced significantly [13].

Human cancers are characterized by an imbalance of regulatory mechanisms controlling different cellular pathways, including apoptosis. Apaf1 and the cysteine proteases known as caspases are central proteins of the intrinsic apoptotic pathway. This pathway of apoptosis is activated by death stimuli that induce the release of proapoptotic factors such as cytochrome *c* from mitochondria into the cytosol and trigger the subsequent formation of the apoptosome, an oligomeric protein complex consisting of Apaf1, procaspase-9, cytochrome *c*, and deoxyadenosine triphosphate (ATP) [17]. Mature caspase-9 activates downstream caspases, such as caspase-3, resulting in the controlled demise of the cell [18]. It is known that the histone acetylation and deacetylation in the retina are epigenetic phenomena that have an influence on the transcriptional repression of *Apaf1* [19]. Furthermore, Tan et al. [20] could show that TSA restores *Apaf1* function in chemoresistant ovarian cancer cells. It is also known that *Apaf1* is inactivated in metastatic melanomas [21]. Interestingly, TSA treatment did not result in increased *Apaf1* levels in melanoma cells, suggesting that HDAC inhibitor effects on apoptotic factors may be cell-type-specific [22]. Moreover, it is known that HDACi can induce p53 acetylation which leads to the transcriptional upregulation of Apaf1 [23]. Moreover, Hanigan et al. [24] could show that HDAC2 regulates *Apaf1* mediating the apoptotic response to HDAC inhibitors.

## Conclusions

Our studies demonstrate that *HDAC1*–*3* play a major role in mediating apoptotic response to HDAC inhibitors through direct regulation of *Apaf1* in HCC.

## Abbreviations

Apaf1: apoptotic protease-activating factor 1
HCC: hepatocellular carcinoma
HDAC: histone deacetylases
HDACi: histone deacetylase inhibitor
qRTPCR: quantitative reverse-transcription polymerase chain reaction
siRNA: small interfering RNA
TSA: trichostatin A
